# Re-Visiting the Embryogenesis of the Human Lower Lip: An Overlooked Paradigm

**DOI:** 10.3389/fphys.2012.00333

**Published:** 2012-08-24

**Authors:** Heleni Vastardis, Meropi N. Spyropoulos, Alphonse R. Burdi

**Affiliations:** ^1^Department of Oral Biology, School of Dentistry, National and Kapodistrian University of AthensAthens, Greece; ^2^Department of Orthodontics, School of Dental Medicine, Tufts UniversityBoston, MA, USA; ^3^Universities of AthensAthens, Greece; ^4^Universities of MichiganAnn Arbor, MI, USA

**Keywords:** human, embryogenesis, lower lip, mandible, bilateral clefts, Pena–Shokeir syndrome

## Abstract

The rare opportunity to study a human fetus showing bilateral clefting of the lower lip along with other associated anomalies resembling those of the equally rare Pena–Shokeir phenotype prompts this report. The scarcity of reports on bilateral clefts of the lower lip has strengthened the conventional understanding or, perhaps even dogma that the lower lip and jaw develop from the progressive midline merging of just two mandibular prominences in the embryo. On the basis of observations stemming from this case report, it is proposed that yet another developmental event or process (in addition to the midline merging of the mandibular prominences) may be operable in the normal morphogenesis of the lower lip and anterior mandibular region. The bilateral paramedian clefting observed provides evidence that another distinct developmental region, a small medial process complements mandibular morphogenesis.

## Introduction

The understanding of the morphology seen in birth defects, the so-called “experiments of nature,” has and continues to expand our knowledge of normal or typical patterns of human morphogenesis. This pertains to the range of birth defects having known or unknown etiologies, or those defects appearing as non-syndromic or syndromic in nature. The latter provide even further opportunities to appreciate the morphogenic associations between different organs in the developing body. The importance of identifying such associations is further strengthened, as one gains an appreciation for the critical parameter of timing in either shaping the normal body or in tracing the perturbations, e.g., teratogens, or unusual genetic expressions, that can alter normal morphogenesis (Moore and Persaud, 1998). The more frequently birth defects occur, the more insight and basic information can become available on patterns of normal morphogenesis. In consequence, since bilateral clefts of the lower lip are of very rare occurrence, our knowledge related to this developmental defect, as well as that of the normal lower lip itself, is also limited, and deserves re-examination. A propos of a human fetus with a rare condition of bilateral cleft of the lower lip showing morphological signs of the even-rarer Pena–Shokeir syndrome phenotype, we revisit the prevailing classical hypotheses related to the development of the lower lip.

## Study Sample

The abnormal specimen featured in this report was received by the Teratology Unit and legally donated to the Department of Anatomy at the University of Michigan, under informed-consent provisions of the State of Michigan’s anatomical donations laws. Received within hours after vaginal delivery, a routine necropsy was performed on the specimen prior to formalin fixation. The specimen is a Caucasian male fetus having a crown-rump length of 225 mm, a crown-heel length of 325 mm, and with no family history of congenital defects. Based on maternal history and the correlative parameters of age (body length, and morphologic characteristics observed), the specimen age was judged as 27 gestational weeks (Arey, 1965; Nishimura et al., 1968; Patten, 1968; Shepard et al., 1988; Moore and Persaud, 1998; Carlson, 1999; Sadler, 2000).

**Figure 1:**
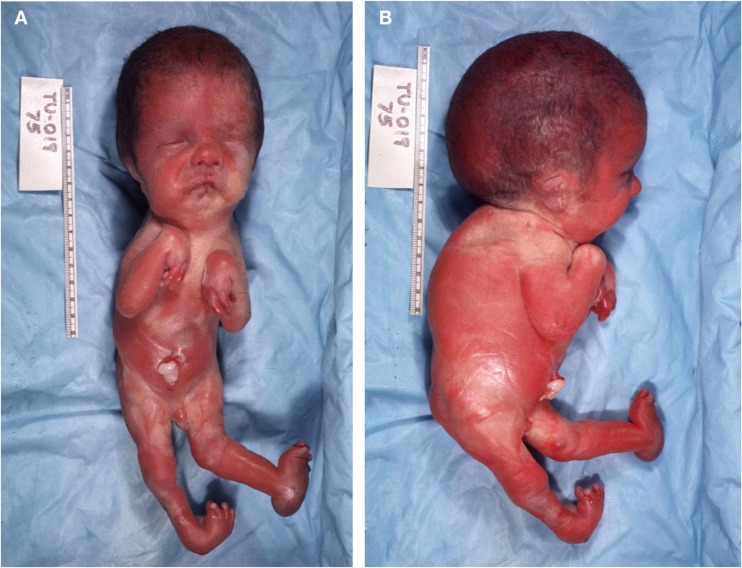
**(A,B)** Frontal **(A)** and lateral **(B)** views of the Pena–Shokeir fetus.

Clinical examination of the craniofacial region (Figures 1A,B) revealed several aberrant macroscopic features as a grossly enlarged head circumference (296 mm); hypertelorism; micrognathia; microglossia; low-set ears; anteverted nares; complete cleft of the palate; bilateral furrows of the lower lip of which the one on the left side was deeper; and a short-thick neck. Postcranially, the specimen exhibited a vertebral kyphosis and rotoscoliosis (curve to right); a short globus penis with underdevelopment (for its developmental age) of scrotal sac; multiple contractures of upper and lower appendicular skeletons; and a deformity of the left foot (calcaneovalgus ankle, rocker bottom, and wide-spaced toes).

Assessment of organs and viscera revealed enlarged brain (99.7 percentile), hypoplastic lungs with abnormal lobations (right side), unfused ventral and dorsal pancreatic elements, and enlarged adrenals. This cluster of defects is suggestive of the Pena–Shokeir syndrome (Pena and Shokeir, 1974).

Using the same reference somatic standards as those used for the Pena–Shokeir resembling specimen, three “typical-for-age” (e.g., “normal”), male caucasian fetuses were selected for age-matched comparative purposes. They were selected from the Patten Embryology Research Collection at the University of Michigan. Specimens were aborted from uneventful pregnancy histories, and represented a comparable age range (230, 253, and 280 C-R lengths; Spyropoulos and Burdi, 2001).

For the specific purpose of comparing prenatal lip and mandibular morphology of the Pena–Shokeir fetus with that of its aged-matched “typical-for-age” specimens, the head of the Pena–Shokeir specimen was removed en-bloc and prepared for routine light microscopy (i.e., neutral-buffered formalin fixation, paraffin-celloidin embedding, serial sectioning at 7 μm; and tri-chrome Mallory connective tissue staining). The three comparative “typical-for-age” fetal specimens were similarly prepared for comparative purposes.

## Histological Findings

**Figure 2:**
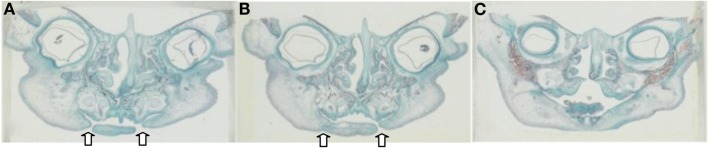
**(A–C)** Representative serial histological sections (frontal plane) from the Pena–Shokeir fetus tracing the progressive merging of the lateral parts of the lip and mandible to the central one (sections from left to right correspond to progression at the A–P direction). Note the two bilateral discontinuations at the lower lip area, one more extensive than the other, both lined with epithelium.

**Figure 3:**
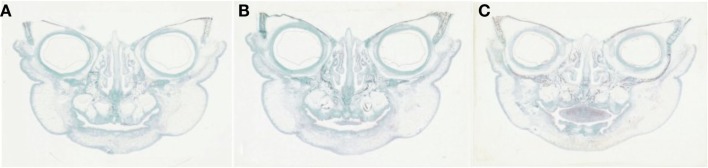
**(A–C)** Comparable serial histological sections (frontal plane) from a “normal” or “typical-for-age” specimen depicting the continuity of the lip and mandibular area throughout the tissues involved, at 27 gestational weeks.

Detailed readings from the serial histological sections, and mapping of the orofacial tissues of the specimens collectively demonstrated a new pattern, or paradigm, of mandibular embryogenesis. With regards to the abnormal fetus (Figures 2A–C): (1) presence of malformation in the nasomaxillary area, with bilateral clefting of the palate and underdevelopment of the tongue and (2) lack of continuity at the anterior lower lip and mandibular area, with two bilateral furrows (pinpointed by arrows), dividing the tissues into three constituent parts, all surface-lined with epithelium. As the serial sections progressed in the antero-posterior direction, progressive merging, or coalescence of the lateral parts with the central-most one was observed, until total continuity was finally established. In comparison, the corresponding serial sections of all three control specimens (one of them presented in Figures 3A–C), showed no evidence of such constituent parts and furrowing, as they showed the existence of complete continuity of the surface contours and inner tissues of the lower lip and anterior mandibular area.

## Discussion

### Prevailing model of the human lip and mandible embryogenesis

The classical picture of lower jaw morphogenesis, taking place between five and seven postconception weeks, shows that there are two bilateral mandibular prominences within the first pharyngeal arch. These prominences separated by a midline surface furrow, enlarge, and essentially come together by the progressive midline merging, or subepithelial filling-out of that midline surface furrow (e.g., Vermeij-Keers, 1990; Moore and Persaud, 1998; Carlson, 1999; Sadler, 2000; Sperber, 2001). The midline dimple or furrow that can be seen in the surface contour of the chin region, in some newborns or older individuals, is considered to be a reflection of the variation in the completeness of merging of the two bilateral embryonic mandibular prominences (Jones, 1988; Gorlin et al., 2001). It is within these consolidated prominences of the lower jaw that the lip, dentoalveolar ridge, mandible, and lower jaw muscles progressively develop beginning at the early fifth postconception week. Toward the fifth and a half postconception week (17-mm CRL embryo), progressive cell differentiation and migration within the embryonic lower jaw give rise to the chin region, lower lip, dentoalveolar processes of the mandible, and muscles (chiefly the orbicularis oris muscle) and mandibular skeletal elements (Spyropoulos, 1977).

Looking specifically at the skeletogenic events that occur in the embryonic chin region as sequelae related to the midline merging of the two mandibular prominences, the anterior-most regions of Meckel’s cartilage progressively ossify to form the bony chin (Ten Cate, 1998). The structural elements in the symphyseal region of the human mandible, including the midline synchondrosis that separates the right and left halves of the future mandible, can be identified in the human embryo as early as seven postconception weeks (25 mm CRL). Development and growth continue in this region throughout the prenatal period, and after birth, through the years of adolescence (Riolo et al., 1974; Farkas, 1994). Disturbances in the morphogenesis and growth of the embryonic symphyseal regions, and more specifically within the anterior-most skeletal regions of the mandible, can result in varying degrees of dysmorphogenesis or even agenesis within the mandibular chin regions that carry over into postnatal life (Warkany, 1971; Jones, 1988; Oostrom et al., 1996; Gorlin et al., 2001).

What has been briefly described thus far, is the classical picture of early embryogenesis (both normal and abnormal) occurring in the midline regions of the cartilaginous skeleton of the lower jaw. Nevertheless, there is another aspect of embryonic mandibular morphogenesis, which remains relatively overshadowed, that of the events occurring in the lip area and off the mandibular midline. Investigation of these events is prompted by our rare Pena–Shokeir fetus.

A closer examination of the developing human embryonic mandibular prominences and future lower lip regions is warranted. The “lip region” is being defined as that region within the lateral limits of the orbicularis oris muscle, or the region between the right and left oral commissures. In as early as 32 postconception days (5–6 mm CRL), four distinct centers of cell differentiation and growth underlie the lower rim of the oral opening, i.e., the anlagen of the future lower lip. Each one of these centers is delineated by a shallow surface groove or furrow. As discussed above, the two anterior-most centers (or surface prominences) are separated in the midline by a median furrow (or intermandibular furrow), which is so-predominantly featured in the embryology literature (Arey, 1965; Carlson, 1999; Gorlin et al., 2001; Avery, 2002). The two lateral centers seem to have been generally overlooked, in spite of the fact that, in some related sketches included in classical textbooks, these centers are clearly shown (Patten, 1968).

Recent investigations in developing chick mandibles provide evidence that mandibular prominences do exist, but consist of independent functional regions, namely, the two large lateral ones and a small medial region (Mina et al., 2002). Functional and tissue distribution studies suggest that different signaling pathways are involved in the morphogenesis of the medial and the lateral regions of the developing chick mandible (Mina, 2001; Mina et al., 2002).

What comes to light now with the fortuitous receipt and follow up study of the rare Pena–Shokeir fetus, is the existence of another set of surface furrows (heretofore unreported in the literature) that provide surface separations of the central prominence of embryonic lower lip and anterior jaw tissues from the lateral set of prominences on either side of the central prominence. Thus, the surface contour of the developing lower lip and jaw region has a single midline furrow (separating the two anterior-most lip prominences) and two lateral – probably shallower – furrows (that separate the two anterior-most from their neighboring lateral lip prominences). By approximately 38 postconception days (10–16 mm CRL), the two lateral furrows are expected to disappear as surface features, through selective, high rate, subsurface proliferation of mesenchymal cells at the base of the furrow. In effect, this selective proliferation of cells gradually pushes the base of the furrow toward the surface with the resultant elimination of the furrow (Burdi and Faist, 1967; Patten, 1968; P. T. Sharpe, personal communication). It has been suggested that disturbances in the development of the mandibular process could result in persistence of the lateral furrows, beyond the 10–16-mm stage embryo, and in the development of congenital fistulae (Warbrick et al., 1952).

A failure in this lateral merging process has been associated with the genesis of lower lip pits (Burdi, 1968). Such pits are usually seen clinically as bilateral and symmetrical depressions or dimples on the vermilion portion of the lip and constitute the most frequently occurring congenital malformation of the lower lip (Watanabe et al., 1951). In other cases, epithelial wall remnants from these lateral grooves may lead to formation of cysts of variable size. Communication between the cyst and a duct of mucous glands may result in a fistula of the lip that may extend deeply into the orbicularis oris muscle. Congenital fistulae of the lower lip usually appear bilaterally on each side of the midline rather than in the middle of the lip (Kitamura, 1989). However, the fact that they can be bipartite in their deepest parts makes it difficult to decide whether they are truly median or paramedian (Rintala and Lahti, 1973). Unilateral lower lip sinuses with or without associated clefts are reviewed in a case report by Ratcliffe and Milling (1989).

Congenital lip pits or fistulae are often associated with other anomalies such as cleft lip and/or palate (van der Woude, 1954; Rintala and Ranta, 1981). It is notable that the grooves disappear at about the same time that fusion occurs between several facial processes. This observation provides a reasonable explanation of why lip pits and clefts may occur simultaneously (Ord and Sowray, 1985).

Upper lip fistulae occur rarely (Ortega-Resinas et al., 1984). From an embryological point of view, their origins are linked with the failure of complete fusion, or apoptosis of the epithelial seams, between the embryonic maxillary and medial nasal prominences, which eventually form much of the upper face (Carlson, 1999).

As noted earlier, bilateral (paramedian) pits and fistulae of the lower lip are rare occurrences, but, when seen, they are usually bilateral and located lateral to the midline on the vermilion border of the lower lip (Cervenka et al., 1967). Reports on lateral, or bilateral clefts of the lower lip are extremely rare; therefore, it is worth reviewing these few cases that resemble the fetus presented here.

Abramson (1952) presented a clinical case in which a newborn Caucasian female infant showed bilateral clefts of the lower lip, along with ankyloglossia, and the presence of a snout-like process protruding downward from the mid-hard palate into the oral cavity. Pertinent to the current report, the bilateral clefts of the lower lip extended deeply through approximately 75% of the lip thickness. In that early report, it was found difficult to explain the embryogenesis of the observed bilateral lower lip clefts based on the view that was firmly entrenched in the literature at the time, which focused exclusively on the midline merging of the furrow separating the two mandibular prominences of the embryonic first pharyngeal arch. Instead, Abramson favored a hypothesis or explanation wherein an “ectodermal hood” covering the oral cavity is invaded progressively by a mesoderm field found within the tissues surrounding the early oral cavity (Cannon et al., 1951). Furthering this hypothesis, the genesis of lower lip clefts was associated to the condition where that “ectodermal hood” was not supported from below due to either a complete or incomplete invasion of the region by the mesodermal field of cells.

Another clinical case showing clefts of the lower lip was reported by Oka et al. (1983) in which a 3-month-old boy presented with a complete bilateral cleft lip and cleft hard and soft palate, a unilateral transverse facial cleft, and a marked paramedian cleft of the lower lip and mandible. The suggested embryogenesis of that case was assigned to a single localized error in morphogenesis of the first pharyngeal arch that could result in such a cluster of defects. Yet, while offering that general suggestion, the authors admittedly found it difficult to use that general suggestion in order to specifically address the embryogenesis of the observed marked paramedian cleft of the lower lip and mandible. Instead, another prevailing hypothesis was favored according to which bilateral clefts of the lower lip and jaw were associated with either interrupted or impeded mesodermal (i.e., mesenchymal) filling-out of the entire first pharyngeal arch both at the arch’s midline and at its lateral extents (Warbrick et al., 1952).

More recently, two cases of a paramedian mandibular cleft accompanied by a variable degree of hemifacial microsomia were described (Morritt et al., 2007; Tanna et al., 2012). While no consensus concerning the embryogenesis of lower facial clefts exists, observations of paramedian mandibular clefts strengthen the arguments of a partial or complete failure of growth center differentiation contributing to the defect (Tanna et al., 2012). Whether this medial mandibular process provides directional outgrowth to the developing mandible becomes worth investigating in hemifacial microsomia cases.

## Concluding Remarks

In this report, the opportunity to study a rare fetus showing bilateral clefting of the lower lip triggered a re-visiting of the embryogenesis of the lower lip. While mindful of the importance of midline merging of the mandibular prominences, observations in this report should draw attention to yet other important merging events that apparently occur, in regions located laterally to the midline, at the sites of the future oral commissures. We have come to contemplate the fact that the lateral grooves mentioned earlier constitute more than serendipitous morphological features with a very short life span. In support to this hypothesis comes the observation that in the aging or aged face in years well-beyond birth, the locale of these embryonic lateral furrows are possibly demarcated as vertical wrinkles seen on either side of the lower lip midline. While complete merging in these lateral lower lip regions results in the smooth surface contour of the lip, partial or incomplete merging may be the pathogenic event associated with lower lip clefts and fistulae, either as isolated defects or as features associated with syndromes like the Pena–Shokeir.

The time-specific effect of such a malformation is undebatable. Might the bilateral furrowing have occurred in normal embryos before the ages we studied, and then disappeared in short time? It is, however, still plausible that a transient but symmetric disturbance in the initial sites of histodifferentiation on the lateral borders of each part of Meckel’s cartilage could potentially account for a bilateral lower lip cleft, as the one reported here for the Pena–Shokeir fetus.

It could also be that, in some cases, the symphyseal cartilages on the two halves of the mandible, do not develop adequately, leaving a defect at the midline area. Therefore, we cannot rule out the possibility that median clefts of the mandible are, in actuality, bilateral due to absence of the middle part of the symphysis. Alternatively, the amniotic band sequence could explain phenotypes such as the Michigan fetus (J. B. Mulliken, personal communication; Jabor and Cronin, 2000). However, such explanation could not support the symmetry in the mandibular defects in contrast to the maxillary ones. The concept that the mandible is a single bulging process formed chiefly by subepithelial neural crest cells that migrate inferiorly from the neural tube to eventually populate the entire mandibular region from both sides, could explain our finding of bilateral mandibular furrows. It appears that the mandibular processes do not fuse in the midline at all. What is rather the case is that flowing masses of migrating neural crest cells fill and “push out” the ectoderm lining of the mandible, to generate what appear to be the two prominences (P. T. Sharpe, personal communication). Dysfunction in other cells likely from the same progenitor cell pool may account for the multi-organ defects.

The significance of observations of bilateral furrowing peculiar to the Pena–Shokeir phenotype goes beyond the descriptive events in mandibular surface features observed. Instead, these observations point to the presence and activity of a gene or a selective set of genes (Kondo et al., 2002) which may determine the patterns of surface contouring and furrowing in the normal and abnormal embryogenesis of the human lower lip. Moreover, the complexity and extent of the fetus phenotype reflect that the mandible specific mutated regulatory genes may be critical for the development of the other affected organs.

## Conflict of Interest Statement

The authors declare that the research was conducted in the absence of any commercial or financial relationships that could be construed as a potential conflict of interest.
